# X-ray Computed Tomography Data of Additive Manufacturing Metrology Testbed (AMMT) Parts: “Overhang Part X4"

**DOI:** 10.6028/jres.125.031

**Published:** 2020-10-01

**Authors:** Max Praniewicz, Brandon Lane, Felix Kim, Christopher Saldana

**Affiliations:** 1 Georgia Institute of Technology, Atlanta, GA 30332, USA; 2 National Institute of Standards and Technology, Gaithersburg, MD 20899, USA

**Keywords:** additive manufacturing, laser powder bed fusion, x-ray computed tomography

## 1 Summary

This document provides details on the data and files generated from post-build X-ray computed tomography (XCT) measurements of the four parts built as part of the “Overhang Part X4” dataset. The “Overhang Part X4” dataset was a three-dimensional (3D) additive manufacturing (AM) build performed on the Additive Manufacturing Metrology Testbed (AMMT) by Ho Yeung and Brandon Lane on June 28, 2019. The files discussed in this document include image sequences for each part, stereolithography files (.STL) of the surface data extracted from XCT. This data is one of a set of “AMMT Process Monitoring Datasets”, as part of the Metrology for Real-Time Monitoring of Additive Manufacturing project at the National Institute of Standards and Technology (NIST). In-situ sensor data, part design, build command and scan strategy data, materials, and associated metadata for this build are described in [1]. Readers should refer to the AMMT datasets web page for updates.

## 2 Data Specifications

**Table:** 

**NIST Operating Unit(s)**	Engineering Laboratory, Intelligent Systems Division
**Format**	Image stacks (TIF), stereolithography file (STL)
**Instrument**	Zeiss Metrotom 800
**Spatial or Temporal Elements**	N/A
**Data Dictionary**	N/A
**Accessibility**	All datasets^i^ submitted to *Journal of Research of NIST* are publicly available.
**License**	https://www.nist.gov/director/licensing

i The National Institute of Standards and Technology (NIST) uses its best efforts to deliver a high-quality copy of the Database and to verify that the data contained therein have been selected on the basis of sound scientific judgment. However, NIST makes no warranties to that effect, and NIST shall not be liable for any damage that may result from errors or omissions in the Database.

## 3 Methods

### 3.1 Experiment and Sample Description

**Fig. 1:**
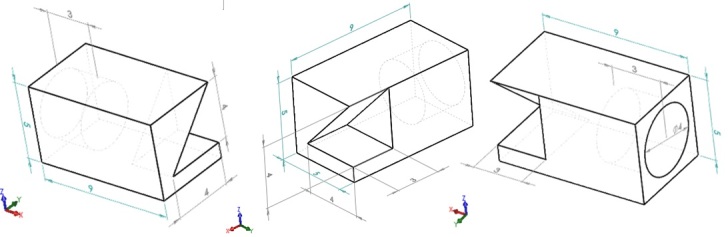
Three views of the external part geometry of the overhang part (units in [mm]). Figure from [1].

The four nominally identical AM fabricated parts discussed in this document were fabricated on the Additive Manufacturing Metrology Testbed (AMMT) from nickel superalloy 625 (IN625). The part geometry, as designed, is a 9 mm × 5 mm × 5 mm rectangular prism with a 45° overhang feature, and horizontal cylindrical cutout. Details on the 3D build, including part layout on the build platform, part design geometry, scan strategy, etc. are described in [1]. For this build, the layer thickness was 20 μm, and nominal process parameters were 195 W power, 800 mm/s scan speed, 100 μm hatch spacing, with D4σ spot size of 60 μm for a Gaussian spot. Scan orientation alternated 0° and 90° between even and odd layers. Figure 1 shows three different view orientations of the computer-aided design (CAD) part geometry. Note the orientation of the part coordinate system, which is consistently applied throughout this document.

After fabrication, each part was electrical discharge machined (EDM) from the build substrate. The EDM processing attempted to cut as close to the substrate surface as possible along the bottom of the part (the XY planar surface in the -Z direction). No additional support structure was included in the build design, and some portion of the part was removed, effectively reducing the 5 mm Z-direction thickness shown in Figure 1. Parts are labelled Part 1 through Part 4, which coincides with the labels in [1] and follows the order in which each part is scanned by the laser within each layer.

### 3.2 XCT Acquisition

**Fig. 2:**
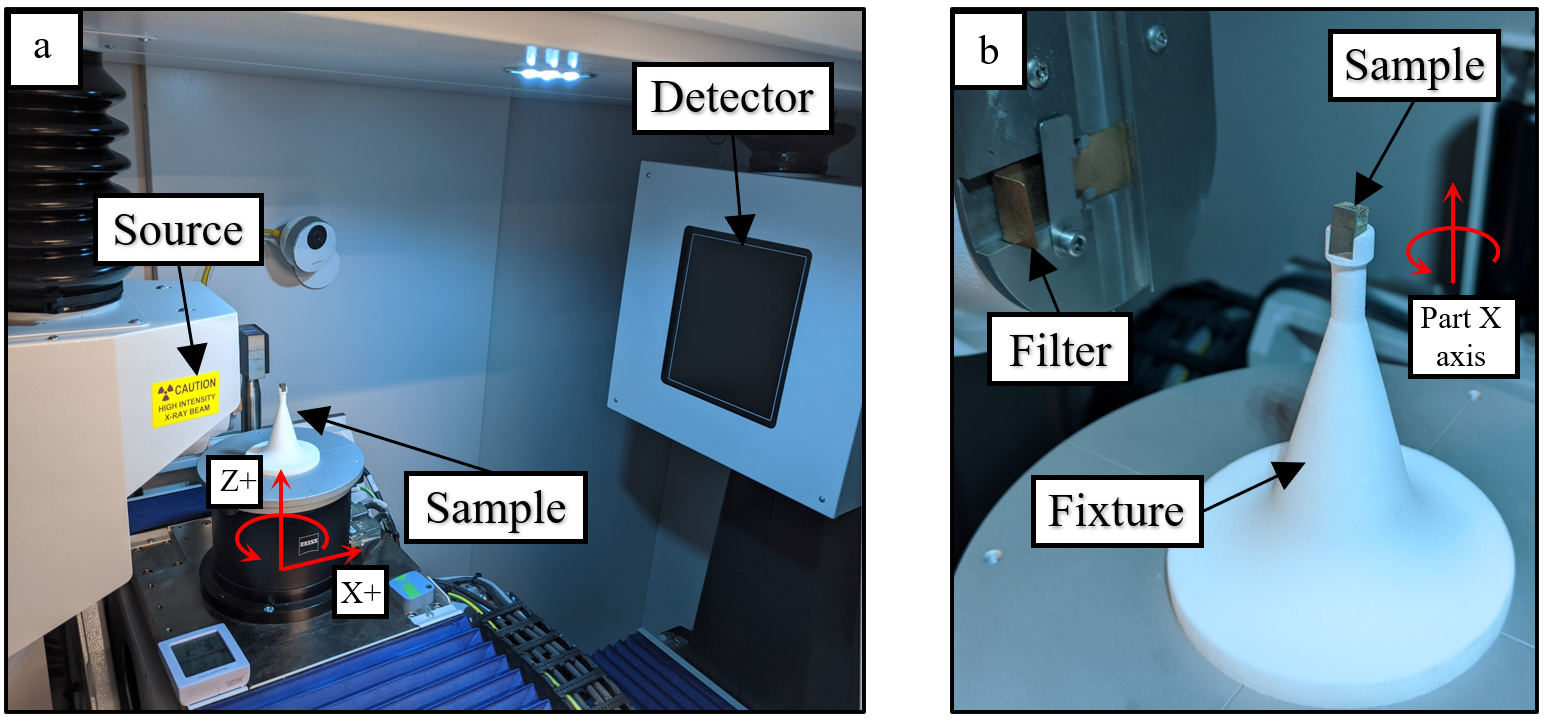
(a) XCT imaging setup and (b) close up of sample. Parts are rotated about the XCT system Z axis, which nominally aligns with the Part X axis.

The parts were scanned on a Zeiss Metrotom 800^ii^ii Certain commercial equipment, instruments, or materials are identified in this paper in order to specify the experimental procedure adequately. Such identification is not intended to imply recommendation or endorsement by NIST, nor is it intended to imply that the materials or equipment identified are necessarily the best available for the purpose.. Each part was inserted into a specially designed fixture to repeatably locate the parts within the measurement volume. This was made from EOS PA 2200 (nylon) in order to reduce the X-ray attenuation of the fixture. The scanning setup can be seen in Figure 2.

Prior to the acquisition of all four scans, all manufacturer-recommended calibration routines were performed. These included a system geometric calibration, a system axis calibration, and a detector calibration. XCT scans for all parts were completed with identical acquisition parameters and took approximately 1 hr and 15 min each. Scans of all four parts were completed successively in order to minimize the variation in environmental effects. The acquisition parameters are listed in Table 1.

**Table 1:** XCT scan parameters.

Parameter	Value
Voltage	130 kV
Current	55 μA
Target (Anode) Material	W
Number of Projections	950
Images Averaged Per Projection	2
Integration Time	1 s
Source to Detector Distance	787.756 mm
Source to Object Distance	60.000 mm
Voxel Size	11.95 μm x 11.95 μm x 11.95 μm
Focal Spot Size	8 μm
Physical Filter	0.5 mm Cu

In addition to these parameters, built in focal spot control was enabled during the course of the scan. Initial measurements of an example sample mounted to the Y axis were conducted at an interval of 64 projections. The results of these measurements are used to detect and compensate for focal spot drift using an interval of 64 rotational steps. Image correction was also applied to each projection. This procedure includes the acquisition of offset images (detector image with X-ray tube switched off with object out of beam path) and gain images (detector image with X-ray tube switched on to default values with object out of beam path) which are then averaged and used to correct the projection. “Homogenization steps” refers to the number of gain images captured at varying intensities. The settings for image correction are listed in Table 2.

**Table 2:** Image correction parameters.

Parameter	Value
Number of images for averaging (Offset image correction)	10
Number of images for averaging (Gain image correction)	10
Homogenization steps	7

**Fig. 3:**
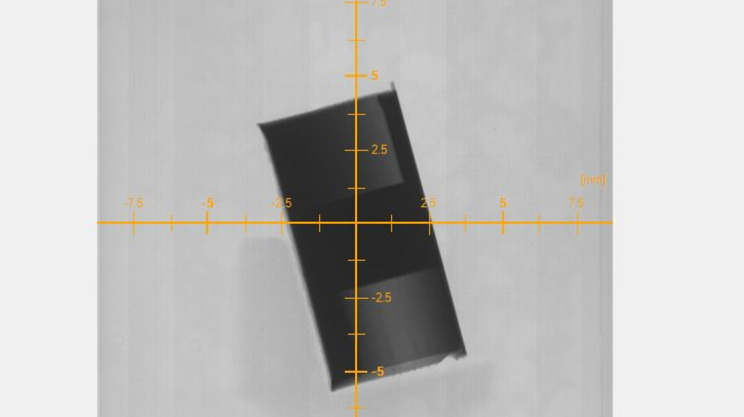
An example radiograph showing Part 1.

Ring artifact compensation was also enabled, which shifted the rotation stage in XCT system Y axis (nominally horizontal in Figure 2) every 10 rotational steps. An example of a completed radiograph is shown in Figure 3.

### 3.3 Reconstruction, Registration, and Resampling

Reconstruction of volumetric data from the individual projection images (radiographs) was completed in Zeiss Metrotom OS with a Feldkamp-David-Kress (FDK) algorithm using a Shepp-Logan digital filter [2]. The reconstructed volume represented as unsigned 16-bit integers was then imported into VGStudioMax3.2 (VG) as a .raw file. In order to fit geometric primitives to the sample for the purpose of registration, a thresholding operation was first performed on the volume. This was completed in two stages. In the first stage, the volume was segmented using an ISO50 method. Then, the surface was refined using the “advanced mode” in VG using a search distance of 8 voxels and was set to remove all particles and voids. This advanced method allows for surface determination at a sub-voxel level, and is similar to other deformable surface algorithms [3–5].

**Fig. 4:**
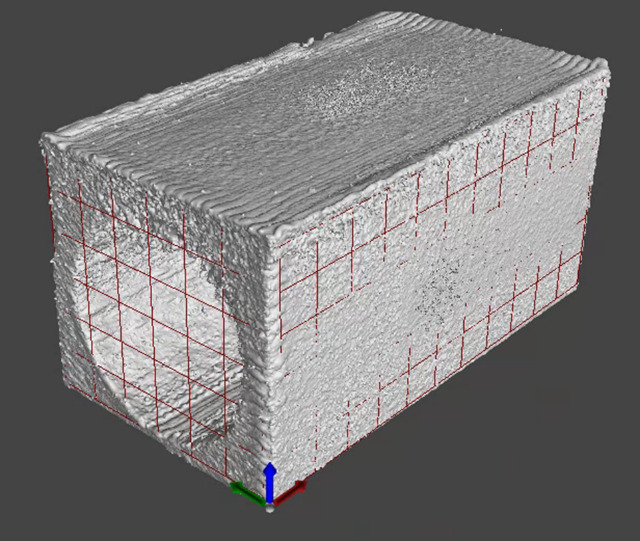
Segmented volume, fit secondary and tertiary datums (primary datum not pictured), and constructed coordinate system for Part 2.

After thresholding, the part coordinate system was determined using a 3-2-1 registration. The part coordinate system was constructed and aligned such that that the orientation of the component matched the orientation during the AM process. The primary datum was chosen as the surface where the part was separated from the platform via EDM. The EDM surface was chosen as the primary datum as opposed to the top surface of the build in order to improve consistency in the fitting of the build axis, as the form errors of the top surface can vary from component to component. The secondary datum was chosen as the -Y oriented face of the component during the AM process. This surface presents the largest area for fitting and allows for greater averaging of small form errors across the four parts. The tertiary datum was chosen as the -X oriented face during the AM process. All datum surfaces were fit to the determined surface using a least squares plane fitting algorithm. The part coordinate system was constructed by sequentially constraining the six degrees of freedom. The plane fit to the primary datum surface first constrained two rotational degrees of freedom and one translational. The interpretation of the secondary datum within the context of the primary datum created a line. This line constrained the remaining rotation degree of freedom and an additional translational degree of freedom. The interpretation of the tertiary datum surface within the context of the primary datum surface followed by the secondary datum created a point. With all six degrees of freedom constrained, a mutually orthogonal part coordinate system was constructed. The XCT data set was then aligned such that the normal vector of the primary datum surface was parallel to -Z, the normal vector of the secondary datum surface was parallel to -Y, and the origin defined by the constrained translation degrees of freedom. Figure 4 shows the segmented volume, fit secondary and tertiary datums (primary datum not pictured), and constructed coordinate system for Part 2.

After all four parts had been registered within VG, the registered volumes were exported relative to the part coordinate system as a .raw volume file and 16-bit TIF image stacks stacked in the part Z direction. These were subsequently processed to crop unnecessary regions and minimize file sizes, discussed in the next section.

## 4 Data Descriptions

### 4.1 Scaled and Cropped TIF Stack Files

**Fig. 5:**
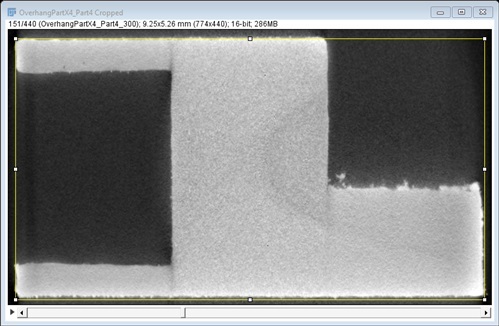
Example TIF stack image (slice 151/440 from Part 4) showing 9.0 mm x 5.0 mm rectangle (yellow).

XCT data is provided as a sequence of 16-bit grayscale TIF images. Scaling is embedded in the TIF image file metadata. Pixel scaling in X and Y is 83.66 pixels/mm, or 0.012 mm/pixel, and physical distance in Z between image slices is also 0.012 mm/slice. Images are cropped to be 774 pixels wide, by 440 pixels tall. With scaling, this results in an image physical width of 9.25 mm wide by 5.25 mm tall. This leaves an approximate 0.25 mm boundary around the exterior of the part, including above and below in Z. Figure 5 shows an example TIF image slice, with a yellow 9.0 mm × 5.0 mm rectangle representing the part design exterior geometry.

TIF image stacks are labeled “OverhangPartX4_PartXX_YYYY.tif”, where XX is the part number and YYYY is the slice index, starting with 0000. The 440 images for each part are compressed into a single zip folder labeled “OverhangPartX4_PartX Cropped.zip”, where XX is part number.

### 4.2 Stereolithography (STL) Surface Files

**Fig. 6:**
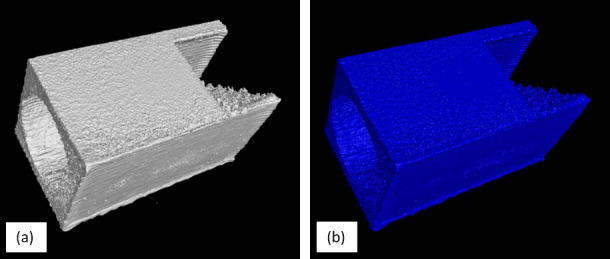
Example 3D visualization of (a) XCT data and (b) corresponding surface mesh.

**Fig. 7:**
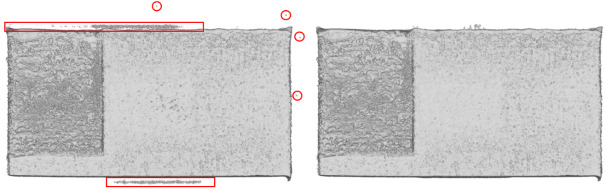
Example mesh cleaning executed on Part 2. The image shows from left to right the STL before and after cleaning.

Prior to export from VG, the determined surface that was defined in section 3.2 was exported as an .STL file. In VG v3.3, a grid-based algorithm was used to create a watertight mesh. The settings for mesh creation included a spatial resampling resolution of 1 voxel and a meshing tolerance of 0.0012 mm. Surface data is exported and labeled “OverhangPartX4 PartX Surface.stl.” While the “remove all particles” setting was enabled in VG, some unconnected triangles were noted which were not connected to the main body of the .STL on all parts. These external triangles were deleted in MeshLab 2020 using the “Remove Isolated pieces (wrt Diameter)” with a maximum diameter of 2.0 mm. These are labeled as “OverhangPartX4 PartX Surface _cleaned.stl.” STL surface files are exported in binary format. Example 3D visualization of XCT data and corresponding surface mesh are shown in Figure 6, and the effect of cleaning is shown in Figure 7.

### 4.3 Notes on XCT Image Artifacts and Features

**Fig. 8:**
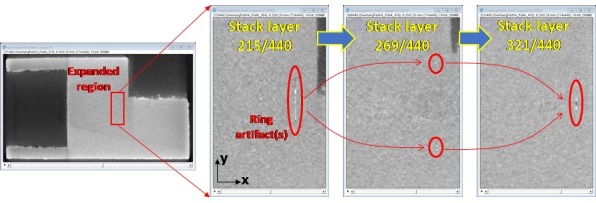
Example residual ring artifacts highlighted in TIF image stacks. Image is from Part 4, and grayscale contrast set between 11130 to 23204 digital levels. Similar features are observed in Parts 1 through 3.

Several measurement artifacts exist that may result in erroneous analyses if proper data pre-conditioning is not considered or attempted. Despite the methods to remove ring artifacts, some still are present within the XCT data, and may erroneously be interpreted as pores. Figure 8 shows an example of three slices/layers from the Part 4 TIF stack, highlighting regions where ring artifacts are still observable. The location(s) and structure of these ring artifacts are similar in all four parts. Users should note these measurement artifacts when conducting thresholding or segmentation to identify pores or defects.

**Fig. 9:**
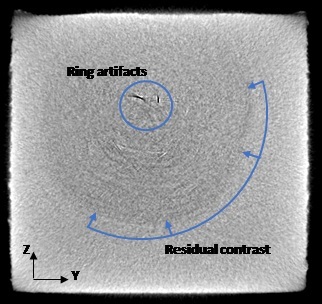
Example YZ planar slice at approximate center of Part 1 showing the circular residual contrast and ring artifacts.

The ring artifacts nominally occur in the YZ plane of each part. Figure 9 shows a YZ-oriented slice of the Part 1 XCT data showing the ring artifacts. Additionally, there is a darker region in the nominal center of the parts, rotationally symmetric about X, resulting a conical shape in the XY plane, and circular shape in the YZ plane.

**Fig. 10:**
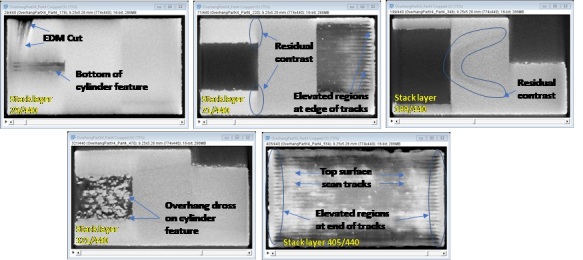
Description of both measurement artifacts and select features observed in the TIF stack images. Image is from Part 4, and grayscale contrast set between 11130 to 23204 digital levels. Similar features are observed in Parts 1 through 3.

To guide users that may be unfamiliar with XCT measurements of AM parts, Figure 10 shows select slices from the Part 4 TIF image stack, and highlights both measurement artifacts (residual contrast artifacts, potentially stemming from the ring-artifact correction procedures), and real structural features on the part. The residual contrast artifacts may be a result of the ring-artifact correction procedures during the measurement, or beam hardening, which occurs due to selective attenuation of the x-ray beam due to variations in the sample geometry and resulting transmission path length through the sample. The features shown may exist over multiple XCT slices, but are generally observable at similar locations among the four parts.

## 5 Data Files

The following files are provided as part of this dataset:

• “OverhangPart_9x5x5mm.STL” – stereolithography file of the as-designed external part dimensions of parts 1 to 4.
• “OverhangPartX4_PartZ Cropped.zip”: Four zipped folders, where ‘Z’ indicates part number 1 to 4.
  o “OverhangPartX4_PartZ_YYYY.tif” - Within each ZIP folder are 440, 16-bit grayscale TIF images, where Z is the part number and YYYY is the slice index, starting with 0000.
• “OverhangPartX4 PartZ Surface_cleaned.zip”: Four zipped files containing stereolithography surface data (STL) extracted from the XCT data, including some artifact cleaning steps. ‘Z’ in the filename indicates the part number 1 to 4.

## 6 Impact

This dataset is supplementary to the in-situ measurement data discussed in [1]. This kind of data is not readily available from commercial AM systems, which may not allow retrieval of machine data, withhold it in proprietary formats, or do not openly disseminate the methods for how that data was acquired. The combination of in-situ and ex-situ AM fabrication data, provided in a well-documented and publicly disseminated format, enables researchers and NIST collaborators to perform a wide range of analyses pertaining to the rapid qualification of AM parts. This is enabled by identifying correlations between process signatures measured in-situ, to part qualities measured ex-situ, through statistical approaches, machine-learning, or other analyses. This dataset is part of the NIST Metrology for Real-Time Monitoring of Additive Manufacturing project.
